# Stability of psychological wellbeing during the COVID-19 pandemic among people with an anthroposophical worldview: the influence of wondering awe and perception of nature as resources

**DOI:** 10.3389/fpubh.2023.1200067

**Published:** 2023-07-21

**Authors:** Anna Steinhausen-Wachowsky, David Martin, Daniela Rodrigues Recchia, Arndt Büssing

**Affiliations:** ^1^Professorship Quality of Life, Spirituality and Coping, Faculty of Health, Witten/Herdecke University, Herdecke, Germany; ^2^Chair of Medical Theory, Integrative and Anthroposophic Medicine, Faculty of Health, Witten/Herdecke University, Herdecke, Germany; ^3^Chair of Research Methods and Statistics in Psychology, Faculty of Health, Witten/Herdecke University, Witten, Germany

**Keywords:** perceived changes, awe, COVID-19 pandemic, wellbeing, coping, anthroposophical worldview, gratitude

## Abstract

**Background:**

During the COVID-19 pandemic, differences in responses and behaviors were observed among specific groups. We aimed to address how people with an anthroposophical worldview behaved with respect to the perception of burden, fears, and wellbeing. As it is an integral part of their lifestyle and convictions, we addressed the influence of wondering awe and gratitude and perception of nature and times of mindful quietness as resources to cope.

**Methods:**

In two cross-sectional surveys with standardized instruments, participants were recruited in 2020 (*n* = 1,252) and 2021 (*n* = 2,273).

**Results:**

Psychological wellbeing was much higher than in other studied groups and populations, with slightly lower scores in 2021 compared to the 2020 sample (Eta^2^ = 0.020), while the perception of the COVID-19-related burden and fear of the future were low in 2020 with a slight increase in 2021 (Eta^2^ = 0.033 and 0.008, respectively). Their transcendence conviction was negatively related to fears of their own infection or the infection of others. Best predictors of their wellbeing were low burden and awe/gratitude, while the best predictors of their burden were low wellbeing and lack of social contacts.

**Conclusion:**

Compared to the general population in Germany, the anthroposophical lifestyle and related convictions may have buffered some of the COVID-19-related burden and helped them to stabilize their psychological wellbeing.

## 1. Introduction

The COVID-19 pandemic posed new challenges for people worldwide. From a scientific point of view, it seems interesting that not only specific groups of people (i.e., occupational groups or social classes) had to cope with an extraordinarily new crisis, but that the entire world population was affected. In addition to fear of disease and death, people were also burdened by social isolation (1), uncertainties, and fears about the future. Social isolation resulted from being cutoff from social contacts and social events and led to loneliness, anxiety, and depression (2–5).

Of course, there were differences in the intensity of perception and engagement with the effects of the pandemic, within a country or a society already due to the social context, health condition, housing situation, or lifestyle of the respective people. Therefore, it makes sense to compare different groups within a society. Studies around the world have investigated whether people had the same, similar, or very different perceptions during the pandemic and what their experiences and behavior were like during the pandemic (6–9).

### 1.1. Positive and negative reactions to a crisis

Scientific interest goes beyond observing perceptions based on the previously mentioned individual criteria of people within a society. The impact of the pandemic allowed an analysis of whether there were much more fundamental differences in response to the pandemic among the heterogeneous world population. In particular, the respective spiritual/cultural realm might have been the cause of different positive or negative reactions and perceptions. Already in the late 1990's, cultural differences in trauma coping were found (10–13), indicating that stressors may change their perceptions and attitudes, making people “stronger” than before. This was described as “post-traumatic growth,” which is a personal development process in terms of reappraisal coping. Post-traumatic growth was observed where the worldview was particularly shaken, that is, where people perceived strong levels of stress and anxiety (14). Thus, in the COVID-19 pandemic, one may ask whether this is fundamentally a “trauma” similar for all people.

There were also individuals who seem to react differently to the experience of crisis, despite being confronted by the same situation (15). One may expect that some people interpreted the stressors differentially or were using either other coping strategies or were more resilient toward the stressors than others. Resilience is described as the innate ability to survive difficult life situations without permanent impairment (16). Interestingly, the research found that there are people who accept a severe crisis as a challenge and try to make the best of the situation with their available resources (10, 17), while others have difficulties to cope.

Thus, the reactions toward the COVID-19 pandemic and its collaterals in a given population are probably a mixture of indifference, burden, trauma, and post-traumatic change. The terms “intra and post pandemic growth” could be used to describe the sum of these pathways, with the addition of those who have simply experienced trauma with no growth.

### 1.2. Different coping strategies of specific groups during the COVID-19 pandemic

During the COVID-19 pandemic, differences in response, behavior, and experience of the crisis event were also observed among specific groups. For example, the Ministry of Ayush in India issued a behavioral plan with daily tasks to strengthen one's immune system and mind (18–20). Whether this was really effective remains a matter of speculation. In Europe, protection by minimizing contact and waiting for a helping medication and/or immunizing vaccination was the core strategy (21, 22).

In a study of Catholic priests from Canada, religious coping style was found to be an important factor in priests' psychological wellbeing, which was low because of the pandemic distress (23). Other groups assumed they were protected against severe courses of corona infection because of their specific lifestyle (15). In the heterogeneous group of yoga practitioners with their specific lifestyle habits and spirituality, 71% regarded themselves as protected against severe courses of a COVID-19 infection because of their yoga lifestyle and practices, particularly those who rejected a vaccination, were younger and more strictly following the ethical principles of yoga traditions (15). Interestingly, their psychological wellbeing was more stable during the pandemic than in the majority of people.

Also, in Seventh-day Adventists, a small free church community with a strict code of ethics and strong cohesion among the parish members, the experience of awe and gratitude had an important mediator effect between spirituality and psychological wellbeing, which remained stable during the pandemic (24). This is a further example of the inner attitude of a circumscribed community with a specific worldview or spirituality to face the pandemic.

Another scientifically interesting group is members of indigenous populations. It was assumed that they would face even greater problems from the pandemic than the rest of the world (25). This was due to the circumstances that it was more difficult for them to get in touch with relevant information and they were not the focus of global aid. So they were on their own, but they also responded with an active coping strategy. For example, there are Indonesian indigenous groups who, through their lived ecocentrism, saw the cause of the pandemic in humanity's misbehavior and tried to restore ecological human–nature relations through rituals and appeals (25).

Comparing these different approaches, one could distinguish quite fundamentally between active and passive coping strategies. While social isolation and waiting for vaccination protection were at the forefront, especially in Western industrialized countries, specific groups and communities with distinct religious convictions and worldviews tended to “go to battle.” They might be convinced that their lifestyle or religious belief (i.e., connecting with higher protecting forces) would protect them against the pandemic and its outcomes, and this could buffer their stress perception and stabilize their psychological wellbeing.

If one concretizes these basic findings, it seems interesting to analyze the behavior and perceptions of respective groups and how this influenced their wellbeing, fears, and concerns during the crisis. The question arises which intra- and post-traumatic changes in attitudes and behavior can be observed. Furthermore, it is of relevance to which resources people may rely on to cope with the pandemic-related restrictions.

### 1.3. Positive attitudes and behaviors to buffer the impact of the restrictions

Even before the pandemic, studies suggested that experiencing nature as a resource had a positive impact on people's wellbeing (26–28). During the pandemic, access to green spaces and the ability to perceive nature as a stabilizing and stress-calming resource were beneficial to cope with the restrictions during the lockdowns (29–31). The opportunity to get outside into nature was a positive factor in resilience to the constraints of the lockdowns (32–34). People used the “extra time” provided by the lockdown for walks in nature, mindful awareness, and more intensive family contacts.

Within the COVID-19 pandemic, studies reported positive health behaviors and more intensive caring for others (6, 33, 35–37), as well as positive changes in experiencing nature, and the importance of relationships with family and friends (6, 33, 37). Experiencing awe and gratitude as non-religious aspects of experiential spirituality were also observed (38, 39). This ability refers to specific moments of pausing and being attracted and emotionally touched by something, resulting in feelings of gratitude. It can be regarded as an ability to resonate with the “sacred” in life, independently from specific religious beliefs (40).

### 1.4. Anthroposophic lifestyle

How did anthroposophists as a circumscribed group of people with a specific worldview and distinct spirituality face the pandemic, as their lifestyle is based on a closer relationship to nature, a strong meditation practice, with a holistic understanding of complementary medicine and lifelong spiritual development (41–43). Although the anthroposophical worldview refers to esoteric issues and has a religious side too, in the Christian Community Church, it does not exclude an affiliation to other religious communities. It is more a lifestyle with an independent medical basis and the conviction that nature is to be considered an immanent being. For anthroposophists, health and illness are seen “holistically” and in interaction with and dependence on the natural, social, and spiritual environment (41, 44). Anthroposophy (in its idealistic teaching) understands life as a path to the development of insight and spiritual capabilities in the service of the world (44).

The 100-year-old anthroposophic medicine defines an independent picture of illness and health in terms of levels of the biological-functional, psychic-autonomous, and spiritual realms. According to its own view, it does not contradict biomedical and natural scientific applications, but intends to expand medical diversity and currently sees itself in terms of integrative medicine (45, 46). Anthroposophic medicine defines the disease, whether functional or pathological, as an imbalance between the so-called “4-fold functions” of the organism. Symptoms are thus the organism's attempt to deal with the pathology. Thus, in the medical understanding of anthroposophy, there is no attempt to suppress a symptom, but the organism is supported with remedies and a positive lifestyle to bring its systems back into balance.

The behavioral and stabilizing resources associated with their way of life (spiritual education, closeness to nature, and strengthening all bodily and soul systems as the basis of health) might thus have helped them to cope with the pandemic-related restrictions. The question, therefore, arises as to whether anthroposophists had less fears and anxiety during the pandemic because of a strong reliance on nature as an immanent reality, and further because of their understanding of health as a holistic system (47, 48) that depends on natural and spiritual factors. Less anxiety would thus mean less stress on the body. This in turn has a positive effect on the immune system (49), which can be a crucial factor in a pandemic. In the understanding of anthroposophy, humans attain valuable stages of development in their lives through experiencing crises, which they may take with them via reincarnation into their next life (which is part of the underlying convictions of some anthroposophists). These convictions can influence how anthroposophists face stressors, illness, and probably also the pandemic.

### 1.5. Aims of the study

In this context, we wanted to investigate how people with an anthroposophic lifestyle perceived the pandemic-related restrictions, particularly their perceived changes, especially in terms of developing positive attitudes and behaviors on the one hand, and fears and worries and thus psychological wellbeing on the other hand. Based on the results of previous studies on the COVID-19 pandemic within a general population (6, 34) and because of the specific spiritual orientation of anthroposophists, we focused particularly on how reference to nature and times of mindful quietness (“silence”) and awe and gratitude as an experiential aspect of spirituality were perceived and what might predict these perceptions. This is because awe/gratitude, in particular, was the best predictor of perceived positive change during the pandemic in a conventional sample of people from Germany (33, 38). In another study, it was shown that it is important how one perceives being out in nature (34) and revealed that people who experienced moments of awe and gratitude in nature were able to protect their wellbeing during the pandemic to some extent.

The role of these perceptions among anthroposophists to buffer the pandemic-related stressors is unclear. They were taken as a specific example of people with distinct worldviews and related attitudes and behaviors.

Therefore, we assume that anthroposophists may have coped differently and were thus more stable during the pandemic and perceived (more) positive changes in attitudes and behaviors because of the pandemic. We do not assume that anthroposophists *per se* have higher wellbeing compared to other people, but that their psychological wellbeing remained more stable during the course of the pandemic as compared to the sharp decline of wellbeing in a more general population (6, 34, 37) because of their underlying convictions and behaviors.

## 2. Materials and methods

### 2.1. Participants

To reach more participants, we chose the snowball sampling technique as a convenience sample for our study. Convenience sampling is a non-probability sampling technique that involves selecting participants based on their accessibility and availability. The information and link to the online questionnaire were distributed via the network of anthroposophists connected to the Goetheanum, Dornach, Switzerland, and private networks of researchers in the field of anthroposophic medicine. Confidentiality was assured and privacy was respected. Participants were recruited in two waves, one from June to October 2020 and the second from August to November 2021. The questionnaire was evaluated anonymously. Neither concrete identifying personal details nor IP addresses were recorded to guarantee anonymity. The study was positively voted on by the ethics committee of Witten/Herdecke University (S-73/2022).

In the first part of the questionnaire, participants could indicate their gender, age, profession, and their own spiritual direction (Anthroposophy, Buddhism, Christianity, Hinduism, Islam, or other). In this evaluation, only participants who indicated anthroposophy as their spiritual direction were included.

### 2.2. Measures

#### 2.2.1. Awe and gratitude

We were interested in examining times of pausing in wondering awe with subsequent feelings of gratitude as an experiential aspect of non-religious spirituality. This was addressed with the 7-item Awe/Gratitude (GrAw-7) scale (50) that has a good internal consistency (Cronbach's α =.82). It uses items such as “I pause and am captivated by the beauty of nature”; “I pause and then think of so many things for which I am truly grateful”; and “In certain places, I become very still and reverent.” The scale captures a person's emotional response to an immediate and overarching perceptual field. Items are rated on a 4-point Likert scale (0—never; 1—rarely; 2—often; 3—regularly) and referenced to a 100-point scale.

#### 2.2.2. Perception of nature and silence

To determine what changes in attitudes, perceptions, and behaviors were perceived by participants due to the COVID-19 pandemic, we relied on the Perception of Change Questionnaire (PCQ) (33). For this study, we used the 4-item subscale Nature/Silence (Cronbach's α = 0.82) (6). Specific items are “I go outdoors much more often”; “I perceive nature more intensely”; “I consciously take more time for silence”; “I enjoy quiet times of reflection”. Items were introduced with the phrase “Due to the current situation...” which referred to the COVID-19 pandemic. Agreement or disagreement was rated on a 5-point scale (0—not at all true; 1—not really true; 2—neither yes nor no; 3—fairly true; 4—very true).

#### 2.2.3. Wellbeing

The WHO-5 WellBeing Index (WHO-5) was used to measure respondents' psychological wellbeing (51). Participants were asked about their wellbeing during the last 14 days. This short scale avoids negatively worded questions. Representative items are as follows: “I have felt cheerful and in good spirits” or “My daily life was filled with things that interest me.” Respondents estimate how often they have had each feeling in the past 2 weeks, with a scale ranging from “never” (0) to “always” (5). Summed values are given from 0 to 25 and 100% values from 0 to 100. Values in the range < 13 (< 50) would indicate decreased wellbeing or even depressive states.

#### 2.2.4. COVID-19-related burden

To measure the negative perceptions due to the restrictions of the pandemic, five questions were presented, for example, perception of being: (1) Restricted in your daily life, (2) Under pressure/stressed, (3) Fearful and Insecure, (4) Loneliness and Social isolation, and (5) Burdened in your financial and economic situation. Answers were measured with five numeric analog scales (NRS), ranging from 0 (not at all) to 100 (very strong). These five variables can be combined into a factor labeled “COVID-19-related burden” (5 NRS) with good internal consistency (33, 50).

From the PCQ, we used two items that address the lack of social contacts (C17) and being connected to friends via digital media (C18). These are related to the perceived burden. Agreement or disagreement was rated on a 5-point scale (0—not at all true; 1—not really true; 2—neither yes nor no; 3—fairly true; 4—very true).

#### 2.2.5. Corona pandemic irritations

We asked the participants about their fears related to the COVID-19 virus infection with two single items [“I am afraid of getting infected” and “I am afraid of infecting friends and/or family” (52)] and one item addressing their point of view regarding the official COVID-19 protection requirements (“I found the strict restrictions on public life in the initial phase of the COVID-19 pandemic exaggerated”). Agreement with these statements was asked on a scale of “not at all—a little—somewhat—very.” From the PCQ, we also used the single item addressing Fear of future (C28). Agreement or disagreement was rated on a 5-point scale (0—not at all true; 1—not really true; 2—neither yes nor no; 3—fairly true; 4—very true).

#### 2.2.6. Transcendence conviction

To address specific convictions (“I am convinced that…”) related to anthroposophic worldview, we used five specific items (“my soul originates in a higher dimension”; “there are higher forces and beings”; “there is rebirth of man (or his soul)”; “influences from previous lives (karma) also have an effect on health and illness”; and “influences from the spiritual world also have an effect on health and illness”) that can be combined to the factor “Transcendence conviction” (Cronbach's α = 0.91). Three of these items were from the ASP questionnaire's Transcendence conviction subscale (53). An additional item from the PCQ addresses trust in a higher supporting power (C32).

### 2.3. Statistical analyses

Descriptive statistics with frequency tables, cross-tabulation (Pearson's Chi^2^) and analyses of variance (ANOVA) of influence and outcome variables, internal consistency (Cronbach's coefficient α), and first-order correlations (Spearman's rho) were calculated using SPSS 28.0. Given the exploratory character of this study, the significance level was set at *p* < 0.01. Group comparisons are reported with *p*-values and effect sizes for better contextualization of results. Here, Eta^2^ values < 0.06 are considered as a small effect, between 0.06 and 0.14 as a moderate effect, and > 0.14 as a strong effect. In classifying the strength of the observed correlations, we considered *r* > 0.5 as a strong correlation, r between 0.3 and 0.5 as a moderate correlation, *r* between 0.2 and 0.3 as a weak correlation, and *r* < 0.2 as negligible or no correlation.

## 3. Results

### 3.1. Description of participants

Participants from two time points were combined in this study: (1) Participants recruited between June and October 2020 (“cohort 1”; *n* = 1,252), that is, a period after the first lockdown in Germany when there were relaxations of restrictions and 2) individuals recruited between August and November 2021 (“cohort 2”; *n* = 2,273), that is, before and during the so-called 4th wave of the pandemic.

These two recruitment waves did not differ significantly in terms of gender. Within both cohorts, more women than men participated (cohort 1: 70.2%; cohort 2: 70.6%). The mean age was almost identical in both cohorts (58.7 ± 12.4 and 58.2 ± 12.3 years, respectively).

When indicating the spiritual direction of life, multiple answers were possible. In both cohorts, the indication of Christianity (64.5% and 64.2%, respectively) and Buddhism (17.3% and 17.7%, respectively) as an additional spiritual direction alongside anthroposophy was stable and strongest. Hinduism (2.9%), Islam (1.0), other (8.4%), and none (2.6%) were of low relevance.

The participants' professions are diverse and range from education, medicine, art therapy, and eurythmy therapy to a large proportion of other professions (Table 1).

**Table 1:** Sociodemographic data of participants.

**Variables**	**Number (%) and mean value (**±**SD)**		***P*-value**
	**Cohort 1 (2020)**	**Cohort 2 (2021)**	
**Gender**			n.s.^b^
Women	879 (70.2%)	1,604 (70.6%)	n.s.
Men	366 (29.2%)	660 (29.0%)	n.s.
Divers/non-binary	7 (0.6%)	9 (0.4%)	n.s.
**Age (years)**	58.67 (±12.41)	58.21 (±12.30)	n.s.^c^
**Profession**			
Administration	73 (5.8%)	128 (5.6%)	n.s.^c^
Economy	64 (5.1%)	141 (6.2%)	n.s.^c^
Artisan	26 (2.1%)	58 (2.5%)	n.s.^c^
Education	308 (24.5%)	633 (27.7%)	0.040^c^
Psychology	56 (4.5%)	116 (5.1%)	n.s.^3^
Medicine	368 (29.3%)	463 (20.2%)	< 0.001^3^
Art therapy	96 (7.6%)	141 (6.2%)	n.s.^c^
Eurythmy therapy	88 (7.0%)	95 (4.2%)	< 0.001^c^
Physiotherapist	38 (3.0%)	71 (3.1%)	n.s.^c^
Other	341 (27.1%)	749 (32.7%)	< 0.001^c^
**Spiritual direction of life** ^a^			
Anthroposophy	1,152 (91.6%)	2,024 (88.5%)	0.004^c^
Buddhism	218 (17.3%)	404 (17.7%)	n.s.^c^
Christian	812 (64.5%)	1,470 (64.2%)	n.s.^c^
Hinduism	36 (2.9%)	85 (3.7%)	n.s.^c^
Islam	12 (1.0%)	32 (1.4%)	n.s.^c^
Other	106 (8.4%)	314 (13.7%)	< 0.001^c^
None	33 (2.6%)	84 (3.7%)	n.s.^c^

a multiple answers were possible.

b Pearson's Chi^2^ test.

c *t*-test.

n.s., not significant.

In cohort 2 (2021), almost one-third of the participants (30.4%) still stated that they had not yet tested for the COVID-19 virus. Altogether, 47% agreed very much that the strict restrictions on public life in the initial phase of the pandemic were exaggerated, 24% agreed somewhat, 11% a bit, and 18% not at all. In the 2020 cohort, 33% agreed very much (27% not at all), and 55% agreed very much (13% not at all) in the 2021 cohort. This increase in the proportion of consenting participants is significant (*p* < 0.001; Chi^2^) and could indicate some kind of resistance attitude to face the outcomes of the pandemic within the group of anthroposophists.

### 3.2. Specific convictions of anthroposophists

At the beginning of the pandemic, 73.2% stated that they have “trust in a higher power” that carries them through difficult times (item C32) (“rather applies” and “applies exactly”). In the second cohort, 58.0% agreed. This loss of trust was significant (*p* < 0.001; Chi^2^).

For 80.7% in cohort 1 and 79.6% in cohort 2, it is “exactly true” that their “soul has its origin in a higher dimension,” and 77.7% vs. 76.3% are convinced (“applies exactly”) that there is “rebirth of the human being or his soul.” When asked whether they were convinced that influences from the “spiritual world” are also at work in the process of health and illness, 91.2% vs. 91.4% answered “rather true” and “true exactly.” For these three statements, there were no significant differences in both cohorts (data not shown).

### 3.3. Psychological wellbeing, COVID-19-related burden, and Fear of future

Psychological wellbeing was relatively high in cohort 1 and was significantly lower in cohort 2; however, this difference is only weak (Table 2). Similarly, the perception of burden was significantly higher in cohort 2 compared with cohort 1; this difference is weak, too (Table 2). Women had lower wellbeing and higher perception of a burden than men, again with a weak effect size. However, the burden was perceived as less relevant in older participants (with a moderate effect size), and also, their wellbeing was better (Table 2).

**Table 2:** Awe/gratitude, nature/silence, and fear of future in the sample.

		**Awe/ gratitude (GrAw-7)**	**Nature/ silence (PCQ)**	**Wellbeing (WHO-5)**	**COVID-19-related Burden (5NRS)**	**Fear of future (C28)**
Range		0–100	0–100	0–25	0–100	0–4
All participants	Mean	69.43	62.68	16.01	27.03	0.57
	SD	16.02	21.31	5.22	20.07	0.96
**Cohorts**						
Cohort 1 (2020)	Mean	70.45	62.83	17.02	22.15	0.46
	SD	16.18	21.88	4.74	17.73	0.82
Cohort 2 (2021)	Mean	68.86	62.60	15.46	29.72	0.64
	SD	15.90	20.99	5.38	20.77	1.02
*F*-value		8.05	0.09	73.55	119.31	28.59
*P*-value		0.005	n.s.	< 0.001	< 0.001	< 0.001
Eta^2^ value		0.002	0.000	0.020	0.033	0.008
**Gender**						
Female	Mean	71.23	64.33	15.67	28.38	0.60
	SD	15.67	21.08	5.25	20.41	0.97
Male	Mean	65.12	58.73	16.81	23.77	0.50
	SD	16.06	21.34	5.04	18.71	0.93
Non-binary/divers	Mean	71.36	60.16	16.69	24.25	1.07
	SD	16.93	21.76	5.24	23.03	1.34
*F*-value		54.56	25.50	17.48	19.52	5.27
*P*-value		< 0.001	< 0.001	< 0.001	< 0.001	0.005
Eta^2^ value		0.030	0.014	0.010	0.011	0.003
**Age cohorts**						
< 41 years	Mean	65.86	56.42	14.09	36.44	0.82
	SD	16.56	23.12	5.07	21.60	1.10
41–50 years	Mean	67.00	59.48	14.58	33.78	0.72
	SD	16.81	22.15	5.47	20.73	1.07
51–60 years	Mean	68.63	61.86	15.45	28.48	0.61
	SD	16.34	20.45	5.32	20.51	0.98
61–70 years	Mean	70.49	64.99	16.86	24.20	0.51
	SD	15.41	20.48	4.97	18.35	0.88
>70 years	Mean	73.06	66.28	17.93	17.49	0.33
	SD	14.72	21.07	4.31	15.22	0.80
*F*-value		16.23	18.16	52.95	**76.93**	18.23
*P*-value		< 0.001	< 0.001	< 0.001	**< 0.001**	< 0.001
Eta^2^ value		0.018	0.020	0.057	**0.081**	0.021

Eta^2^ values < 0.06 are considered as a small effect, between 0.06 and 0.14 as a moderate effect, and >0.14 as a strong effect.

In cohort 1, 85% stated that it was not at all true or rather not true that they were afraid of the future. In cohort 2, 80.1% also answered this way. The mean score for fear of future was significantly different, but not relevant (Table 2).

Further, 88% of the participants in cohort 1 had no or little fear of contracting COVID-19, while it slightly increased to 91% in cohort 2 (data not shown). Also, when asked whether they were afraid of infecting friends or family members, 74% answered not at all or a little in cohort 1 and 81% in cohort 2) (data not shown). These convictions may have contributed to having less fears and to their more stable psychological wellbeing.

### 3.4. Experience of nature/silence and awe/gratitude

92.0% of respondents in cohort 1 stated that they often or very often “stopped and were spellbound by the beauty of nature.” This value remained stable in cohort 2 as well (92.9%). 88.9% stated that they were often or very often overcome by a feeling of great gratitude. In cohort 2, these values continued to score high at 87.5%. Likewise, an overwhelming feeling of wondering awe was felt by 77.3% in cohort 1 and by 71.9% in cohort 2. The awe/gratitude score was significantly lower in cohort 2, but this difference is not relevant (Table 2).

About half of the anthroposophists surveyed went outside into nature in both waves (50.2% in cohort 1, 49.5% in cohort 2). The question of whether they perceive nature more intensively was answered by 61.6% with “rather true” or “true exactly.” This value also remained stable in cohort 2 (59.0%). In cohort 1, 36.3% of participants stated that they took neither more nor less time for periods of silence, while 44.4% took more time for silence. This score remained almost identical in cohort 2: 37.6% of respondents noted no change in their behavior, and 44.7% consciously took more time for silence. In total, 55.9% enjoyed quiet periods of reflection. This score also remained stable during the second cohort (54.1%). The resulting nature/silence scores did not differ in both cohorts (Table 2).

What about their social contact? In cohort 1, 27.7% stated that they lack social contacts (51.1% disagree and 21.3% are undecided), while in cohort 2, 33.4% stated that they lack social contacts (43.9% disagree and 22.7% are undecided). This increase is weak but significant (*p* < 0.001; Chi^2^). In cohort 1, 44.5% stated that they were connected with friends via social media (29.9% disagree and 25.5% are undecided), while in cohort 2, 43.44% were connected via digital media (30.7% disagree and 25.9% are undecided). This decrease is in trend only remarkable (*p* = 0.026; Chi^2^).

### 3.5. Correlations between wellbeing and burden, awe/gratitude and nature/silence, transcendence conviction and fears

We were particularly interested in anthroposophists' perceptions of awe/gratitude and nature/silence and the relationship of these resources to psychological wellbeing and COVID-19-related burden on the one hand and their fears on the other hand.

As shown in Table 3, awe/gratitude and nature/silence were moderately interrelated. While awe/gratitude was weakly associated with wellbeing, nature/silence is only marginally related. Both resources were not relevantly associated with the COVID-19-related burden. Fear of the future was weakly associated with low wellbeing and with burden, and marginally only with awe/gratitude and nature/silence.

**Table 3:** Correlations between wellbeing and burden, awe/gratitude and nature/silence, and transcendence conviction and fears.

	**Awe/ gratitude (GrAw-7)**	**Nature/ silence (PCQ)**	**Wellbeing (WHO-5)**	**COVID-19-related burden (5NRS)**	**Transcendence conviction (ASP+)**
**Awe/gratitude**	1.000				
Nature/Silence	0.318^**^	1.000			
Wellbeing	0.292^**^	0.141^**^	1.000		
COVID-19-related Burden	−0.137^**^	−0.029	−0.573^**^	1.000	
Transcendence conviction	0.233^**^	0.142^**^	0.131^**^	−0.110^**^	1.000
Lack of social contacts	−0.108^**^	−0.047^**^	−0.328^**^	0.449^**^	−0.106^**^
Connected to friends via digital media	0.062^**^	0.142^**^	0.044^**^	0.125^**^	−0.044^**^
Fear of the future	−0.160^**^	−0.053^**^	−0.225^**^	0.270^**^	−0.284^**^
Afraid of getting infected	−0.074^**^	0.035	−0.040	0.034	−0.210^**^
Afraid of infecting friends or family	−0.076^**^	0.027	−0.054^**^	0.025	−0.225^**^
Strict restrictions in the initial phase of the pandemic were exaggerated	0.042	0.012	−0.019^**^	0.209^**^	0.206^**^

** *p* < 0.001 (Spearman's rho).

Lack of social contacts was moderately related to (low) psychological wellbeing and COVID-19-related burden. In contrast, being connected to friends via digital media was marginally only related to COVID-19-related burden and nature/silence (Table 3).

Fear of contracting the COVID-19 virus or being afraid of infecting friends or family was either not or only marginally related to the tested variables (Table 3).

Participants' transcendence conviction was at least weakly related to awe/gratitude, and marginally only to nature/silence, wellbeing, and burden (Table 3). When their transcendence conviction is high, they have lower fear of the future, are less afraid of getting infected and of infecting others, and are more convinced that the strict restrictions in the initial phase of the pandemic were exaggerated, and vice versa (Table 3). This indicated that this conviction might be a relevant buffer for them.

### 3.6. Predictors of psychological wellbeing and COVID-19-related burden

What are the reasons that participants' wellbeing was so stable during the pandemic? To address this, two stepwise regression analyses with either wellbeing (Table 4) or COVID-19-related burden (Table 5) as dependent variables were performed. As we assume awe/gratitude and nature/silence as resources, and fears of future and lack of social contacts as stressors, while also their transcendence conviction may influence wellbeing, these were included as independent variables.

**Table 4:** Predictors of psychological wellbeing (stepwise regression analyses).

**Dependent variable: Wellbeing (WHO-5)** **Model 8: *F* = 311.6, *p* < 0.001; *R*^2^ = 0.43**	**Beta**	***T***	***p***
**(Constant)**		20.815	< 0.001
COVID-19-related burden	−0.503	−31.338	< 0.001
Awe/gratitude	0.206	14.438	< 0.001
Male gender	0.086	6.371	< 0.001
Nature/silence	0.072	5.133	< 0.001
Lack of social contacts	−0.075	−5.014	< 0.001
Age cohorts	0.040	2.857	0.004
Year of recruitment (cohorts 1 and 2)	−0.034	−2.555	0.011
Fear of future	−0.032	−2.287	0.022

Not significant in the model: Strict restrictions were exaggerated and AM Spirituality.

**Table 5:** Predictors of COVID-19-related burden (stepwise regression analyses).

**Dependent variable: burden (5NRS)** **Model 9: *F* = 354.9, *p* < 0.001; *R*^2^ = 0.49**	**Beta**	***T***	***p***
**(Constant)**		21.975	< 0.001
Wellbeing (WHO-5)	−0.447	−30.911	< 0.001
Lack of social contacts	0.266	19.871	< 0.001
Age cohorts	−0.147	−11.357	< 0.001
Strict restrictions were exaggerated	0.093	7.223	< 0.001
Fear of future	0.112	8.549	< 0.001
Awe/gratitude	0.056	4.012	< 0.001
Year of recruitment (cohorts 1 and 2)	0.064	4.985	< 0.001
Nature/silence	0.048	3.631	< 0.001
Male gender	−0.029	−2.269	0.023

Not significant in the model: AM Spirituality.

In the eight-step regression model, wellbeing as a dependent variable was best explained by the COVID-19-related burden (36% variance explanation), with a further 5% added by awe/gratitude, while male gender, nature/silence, lack of social contacts, age cohorts, recruitment cohorts, and fear of future, altogether would add only 2% of additionally explained variance, and are thus less relevant. Transcendence conviction was not relevant as an independent predictor of psychological wellbeing.

In the nine-step regression model to explain the COVID-19-related burden, lack of psychological wellbeing was the best predictor, explaining 36% of the variance. Lack of social contacts would add 7% of explained variance, and age cohorts further 2.5% of explained variance. The perceptions that the strict restrictions were exaggerated, fear of future, awe/gratitude, year of recruitment, nature/silence, and male gender altogether would add only 3.5% of additionally explained variance, and are thus less relevant. Transcendence conviction was not relevant as predictor in this model.

## 4. Discussion

We were interested in examining the convictions, attitudes, and behaviors of people with anthroposophic lifestyles during the pandemic with regard to perceptions of nature, moments of silence and reflection, and awe and gratitude as parts of their concept of life, and their relation to psychological wellbeing and perceived burden.

We found that wellbeing was quite similar in both cohorts. Even when their wellbeing was slightly lower in cohort 2 compared with cohort 1, it is nevertheless higher compared to a reference sample from similar phases of the pandemic (34, 37), while participants' perception of burden was higher in cohort 2. This means that the pattern found in the general population is similar in principle but less pronounced. In addition, the significant decline of “trust in a higher power” as a reliable resource of hope found in this study can be found in the general population (54).

Changes in terms of their utilization of nature as a resource and sometimes to reflect because of the pandemic were observed within the sample, too, and these resources remained stable in both cohorts. Moments of wondering awe and gratitude scored high in cohort 1 and were slightly lower in cohort 2, but not relevantly. This stability of resources can be regarded as relevant. Connecting to nature and enjoying contemplative and quiet moments has been repeatedly studied as a therapeutic element in recent years (26, 55) and highlighted as resources (37). Particularly, awe/gratitude is relevantly associated with psychological wellbeing but seems not to buffer the COVID-19-related burden and fears. Both, the resource and the resulting outcome remained quite stable in this sample. During the pandemic, a remarkable proportion lacked social contacts (28% and 33%) and they stated to be connected with friends also via digital media (45% and 43%). This lack of social contact was a relevant predictor of their perceived burden, both not for their wellbeing.

An interesting finding of our study was that anthroposophists had little to no fear of contracting the COVID-19 virus throughout the pandemic or of infecting friends or family members. In addition, at the beginning of the pandemic, 97% of participants reported having no fear of the future; these statements marginally decreased in cohort 2. Thus, most participants did not regard the pandemic-related burden as a “trauma” in general as they obviously were able to cope with these pandemic-related outcomes and fears. Fears and perception of burden were slightly higher in women compared with men, and lower in older participants compared with younger participants. These (implicit) convictions of being rather protected or resistant may have contributed to having less fears and to their more stable psychological wellbeing. In fact, their transcendence conviction was related to lower fear of the future, of being less afraid of being infected and of infecting others, and that the strict restrictions in the initial phase of the pandemic were exaggerated. This indicated that this conviction (whether it is really true or not) might be a relevant buffer for them.

As we assume that the anthroposophic lifestyle with its specific convictions might contribute to the observed effects, it is important to underline that transcendence conviction is in fact related to participants' age. It is highest in the older participants, with a weak effect size (Eta^2^ = 0.043; *p* < 0.0001). As stated, it might be a buffer against fear and made them mentally more resistant. It is further related to awe/gratitude as an experiential aspect of spirituality, but marginally only to nature/silence which was primarily assumed as highly relevant for people with an anthroposophic worldview. We nevertheless cannot exclude the possibility that their transcendence convictions are rather ideals than lived reality and that these ideals have only little to do with concrete behaviors and attitudes. This would be further underlined as it contributes nothing to the regression model to explain participants' psychological wellbeing. This was exclusively explained by the low perception of the COVID-19-related burden, with a further influence of awe/gratitude, while nature/silence or fears of the future were in fact of minor relevance.

It might be that participants' specific lifestyles and related spiritual convictions could have played a stabilizing role here and protected them against pandemic-related anxiety. Their perception of the COVID-19-related burden was quite low. It can be explained best by their psychological wellbeing, which was relatively high and stable, and further by their relatively low fear of the future. Presumably, the anthroposophists did not feel shaken by the restriction of their ability to act. Thus, trauma-induced growth in the sense of Park et al. (56), Cann et al. (14) and Mangelsdorf and Eid (57) cannot easily be assumed because there is no indication of significant burden or ‘trauma'. Instead, the more intense perception of awe and gratitude can be regarded as an experiential resource (among others) that stabilizes their wellbeing. A further resource seems to be their (cognitive) transcendence convictions that could be assumed to buffer some of their fears (explaining at least 5% of variance), but it cannot buffer the pandemic-related burden or contribute to their wellbeing.

According to Tedeschi and Calhoun (10), the perception of positive changes in a crisis may be an intrapsychic response that people use to protect themselves in a crisis situation. Studies have shown that perceived positive changes because of the pandemic are not necessarily associated with high wellbeing (6). Thus, perceived changes appear to be an independent quality in people's lives that allows them to focus on what continues to be positive in life. This is consistent with findings that positive and negative perceived changes were not correlated following a crisis experience (14).

### 4.1. Limitations

In the first cohort recruited between June and October 2020, *n* = 1,252 people participated, while in the second cohort recruited between August and November 2021, *n* = 2,273 people participated. Because of the recruitment process via the distinct research networks and the subsequent snowball sampling approach, we had no control over the recruitment processes. Since the data collection was an online survey, it could be that people who do not have internet access were not reached. The sample could be of limited representativeness for all anthroposophists, as it was only sent out in the Goetheanum Newsletter and passed on from there via snowball sampling.

The data of this study refer to two cohorts at two characteristic phases of the pandemic, and it is thus not comparable to data from a longitudinal study with the exact same participants. This may have an influence on the data. However, there are no relevant differences in the social-demographic characteristic of participants, and thus, we assume that their perceptions and attitudes refer to the respective phase of the pandemic rather than to changes in participant characteristics.

We cannot exclude that the response was overall positive in terms of social desirability and also positive self-concepts.

## 5. Conclusion

Considering the aforementioned limitation that the findings are not from a longitudinal study with the same participants, but from two cohorts with similar sociodemographic characteristics, we conclude that the anthroposophic lifestyle and related convictions may have contributed to making them more robust against the COVID-19-related fears and worries and helped them stabilize their psychological wellbeing, which was much higher than in other cohorts in Germany. Experiences of nature, wondering awe and gratitude, and spirituality already seemed to have been relevant in their lives even before the onset of the pandemic, and thus, they could have activated these more easily as resources. Thus, these stable perceptions and behaviors during the crisis suggest that an already established concept of life could buffer some of the crisis-related fears and worries.

Awe and gratitude even during a crisis and seeking out natural spaces as a refuge for silence and contemplation can provide a sense of security (26, 34, 58). In the future, prevention programs could address these aspects of active coping, as awareness of inner-psychic resources strengthens feelings of agency. Spiritual exercises and awareness (59), mindful moments of standing in awe and gratitude as core values (40, 60, 61), as well as didactic concepts such as nature bathing (28, 62, 63), could be taught to people in order to provide them with resources to cope with future (also pandemic)-related stressors. It seems to be important to learn from small groups with strong cohesion and specific convictions that influence their attitudes and behaviors.

## Data availability statement

According to the data protection regulations, the data set cannot be made publicly available. Data are however available from the first author upon reasonable request.

## Ethics statement

The studies involving human participants were reviewed and approved by Ethics Committee of the Witten/Herdecke University (S-73/2022). Written informed consent for participation was not required for this study in accordance with the national legislation and the institutional requirements.

## Author contributions

AB designed the study, set up the online survey, and initiated the sampling processes. DM proposed the project, worked on the questions, and provided resources. AB and DR undertook statistical analyses. AS-W and AB wrote the first draft of the manuscript. AS-W, AB, DR, and DM provided feedback and approved the final manuscript. All authors contributed to the article and approved the submitted version.
